# Advanced gesture recognition in Indian sign language using a synergistic combination of YOLOv10 with Swin Transformer model

**DOI:** 10.1038/s41598-025-18496-8

**Published:** 2025-09-25

**Authors:** Umang Rastogi, Rajendra Prasad Mahapatra, Sushil Kumar

**Affiliations:** 1 Department of Computer Science and Engineering, SRM Institute Science and Technology Delhi-NCR Campus, Delhi-Meerut Road, Ghaziabad, 201204 Uttar Pradesh India; 2 Department of Computer Science and Engineering Data Science & Deep Learning Lab, KIET Group of Institutions Ghaziabad, Ghaziabad, 201206 Uttar Pradesh India

**Keywords:** Indian sign language, Indian sign language recognition, YOLOv10, YOLOv10-ST, Deep learning, Computational science, Computer science

## Abstract

Communication between deaf or mute individuals and hearing persons is often hindered by the lack of mutual understanding of sign or vocal language. To bridge this gap, Indian Sign Language Recognition (ISLR) systems are essential. This paper proposes a real-time ISLR framework based on the YOLOv10-ST model, which integrates the Swin Transformer into the YOLOv10 architecture for enhanced feature extraction. The model also incorporates Mish activation to improve gradient flow and detection accuracy. A custom dataset comprising 15, 000 static images (1, 000 per sign for 15 signs) and 35 dynamic videos (covering 7 sign classes) was used for training and evaluation. Experimental results demonstrate high performance, with the model achieving 97.50% precision, 98.10% recall, and 96.58% F1-score for image-based sign recognition, and 95.24% precision, 96.00% recall, and 95.87% F1-score for video-based gestures. The model also achieves a mean Average Precision (mAP) of 97.62% and real-time inference speeds of 48.7 FPS. Ablation studies validate the contributions of Swin Transformer and Mish activation, while paired t-tests confirm statistical significance (p $< 0.005$). The experimental findings demonstrate that the YOLOv10-ST model efficiently recognizes static and dynamic ISL in real time with minimal computational overhead.

## Introduction

Deaf people utilize sign language as one of their nonverbal modes of communication. Understanding of sign language by normal people is complicated, which leads to the barrier of communication with deaf people. When deaf or dumb individuals use sign language to communicate with one another in real life, a problem develops when they attempt to converse with non-deaf individuals^1^. It is also observed that the sign languages in India have their own vocabulary and grammatical structures^2^. In order to understand the meaning of signs used by users to communicate verbally, the Sign Language Recognition (SLR) system simulates the role of a translator. According to Indian Sign Language Research and Training Centre (ISLRTC), for the 7 million hearing-impaired and deaf individuals in India, only 300 competent Indian Sign Language (ISL) interpreters are available^3^. Indian Sign Language Recognition (ISLR) faces numerous challenges, such as variations in hand gestures due to differences in size, shape, skin tone, and orientation of the hands among individuals^4^. Another critical issue is the dynamic nature of ISL, which involves continuous motion and requires systems to process sequential data effectively^5^. Illumination variations, occlusions, and background noise in real-time environments further complicate gesture recognition. Moreover, ISL recognition systems need the simultaneous use of both hands in many signs, which adds complexity to the task. Existing methods often suffer from low accuracy, reduced speed, and an inability to generalize effectively across diverse datasets, making real-time applications difficult^6–8^. The main aim is to develop ISLR system that processes the image/video data containing input signs and provides information about Indian signs. There are various methodologies proposed in the literature^9–13^ to develop this type of system. To detect the signs in image/video, several versions of You Only Look Once (YOLO) based on deep neural networks are proposed to reduce the recognition time^14–19^, but face challenges with small objects, localization accuracy, fixed grid limitations, generalization, and computational efficiency, especially in complex or edge-case scenarios.

YOLO has evolved as a popular object detection framework due to its real-time performance and high accuracy. ISLR using hand gestures has seen significant advancements with the application of various YOLO versions. YOLOv1 introduced real-time object detection with impressive speed and accuracy, laying the foundation for gesture recognition by detecting hand regions^20^. YOLOv2 and YOLOv3 improved accuracy and multi-scale detection capabilities, making them more suitable for recognizing complex hand gestures^21,22^. YOLOv4 brought further enhancements in speed and precision with techniques like CSPDarknet and Mosaic augmentation, boosting real-time ISL recognition^23^. YOLOv5, widely adopted due to its lightweight architecture and faster inference, was pivotal in improving recognition efficiency^24^. YOLOv6, YOLOv7, and YOLOv8 focused on optimizing deep learning models, further improving detection accuracy and adaptability to dynamic hand movements^25–27^. YOLOv9 and YOLOv10, though relatively new, integrate cutting-edge AI techniques, offering unprecedented accuracy and efficiency for ISL recognition, even in challenging scenarios like occlusion or background clutter^20^. Each YOLO iteration has progressively improved real-time hand gesture recognition for ISL, contributing significantly to human-computer interaction and accessibility technologies. When YOLOv10 was initially applied for ISLR using the Darknet backbone, several limitations were observed. Darknet is an extremely effective deep learning framework, however, it is not suitable for lightweight or real-time applications such as mobile phones, small devices and edge devices like cameras and IoT devices. It is not efficient for fast recognition as its internal structure is deep and complex requiring a significant amount of processing time for input such as an image or video. Additionally, the feature extraction capability of Darknet was not as efficient in capturing fine-grained details, particularly under challenging conditions like occlusions or complex backgrounds. To address these issues in YOLOv10, the Darknet backbone is replaced with the Swin Transformer (ST) architecture called YOLOv10-ST. The lightweight design of ST significantly reduces computational overhead, enabling faster inference. Its advanced feature extraction capabilities improve accuracy by effectively distinguishing intricate patterns in dynamic and static gestures and replacing traditional activation functions (e.g., Linear and ReLU) with the advanced activation function is Mish, for better gradient flow and performance. By incorporating this modification, the YOLOv10 model demonstrates enhanced overall performance, making it more suitable for real-time applications, particularly on resource-constrained devices. This paper makes the following key contributions in response to the limitations of prior ISL recognition research:

- **YOLOv10-ST:** We propose a novel architecture that integrates Swin Transformer blocks into YOLOv10. Unlike traditional YOLO versions that rely on CNNs^28^, our model captures both local features and global dependencies through hierarchical self-attention, improving the recognition of visually similar ISL gestures.
- **Real-time, multi-scale detection:** The model leverages v10Detect, decoupled heads, and a multi-scale fusion strategy (SPPF + PSA), resulting in both high accuracy and real-time performance. It achieves 97.62% mAP and 48.7 FPS on image data, outperforming YOLOv3-YOLOv10 baselines.
- **Context-aware architecture:** By eliminating the reliance on Non-Maximum Suppression (NMS) and using one-to-many label assignment, YOLOv10-ST addresses the issue of gesture overlap and occlusion more effectively than prior YOLO variants.
- **Diverse dataset:** A private dataset of ISL gestures is constructed using images and videos under diverse lighting conditions, backgrounds, and from multiple participants. This enhances the generalizability of the model, unlike previous works that relied on more constrained datasets.
- **Statistical validation:** The proposed model’s superiority is not only shown through standard metrics but is also supported by a paired t-test, confirming statistically significant performance improvements over YOLOv10.

The organization of the rest of the sections is as follows: Section “Related Work” presents a review of recent studies in the domain of sign language recognition, particularly focused on Indian Sign Language (ISL), object detection frameworks, YOLO-based models, and transformer-based approaches. After that, Section “Methodology” describes the experimental setup, dataset details, participant demographics, environmental conditions, annotation process, training/testing protocol, consent procedures, and ethical considerations. Section “Proposed Work” provides a detailed explanation of the YOLOv10-ST architecture, including its integration of the Swin Transformer, Mish activation, and improvements over previous YOLO versions. Later on Section “Experimental Results and Discussion” presents a comprehensive evaluation of the model’s performance, including result analysis, statistical validation, ablation studies, real-time inference analysis, and comparisons with both YOLO variants and transformer-based detectors. The last Section “Conclusion and Future Directions” concludes the paper by summarizing the key findings, acknowledging current limitations, and outlining future research directions, including dataset expansion, integration of explainability tools, and ethical compliance.

## Related work

Sign Language Recognition (SLR) involves the complex interpretation of hand shapes, movements, orientations, facial expressions, and body posture. This complexity is compounded by signer variability, occlusions, background clutter, and lighting conditions^29^. Initial research in SLR, including Indian Sign Language (ISL), relied heavily on traditional machine learning techniques and handcrafted features like SIFT and HOG^30,31^. These methods struggled with dynamic gestures and lacked robustness in variable real-world settings^32^. Challenges also included the inability to model complex spatiotemporal dependencies and over-reliance on manual feature engineering^33–35^. Modern deep learning (DL) approaches such as Convolutional Neural Networks (CNNs) and Recurrent Neural Networks (RNNs) overcame many ML limitations by learning spatial and temporal features directly from raw input^28,36–38^. DL’s popularity surged due to improved accuracy and availability of high-performance GPUs^39,40^. Researchers have also used multimodal techniques that incorporate hand keypoints, facial cues, and body posture to enhance recognition^41,42^. However, standardized, large-scale ISL datasets remain limited^43^, affecting generalization. YOLO models (e.g., YOLOv4 to YOLOv9) have shown high speed and reasonable accuracy for gesture detection in videos^44,45^. These models predict bounding boxes and class labels in a single forward pass, making them suitable for real-time applications. Despite improvements in detection heads and feature extractors, these CNN-based YOLO variants still fall short in capturing global attention or temporal dependencies critical for dynamic signs. Transformer models such as ViT and Swin Transformer are increasingly used for SLR due to their ability to model long-range dependencies and hierarchical attention^46,47^. When combined with CNNs, they enhance spatial and temporal context understanding^48,49^. Additional techniques such as Temporal Convolutional Networks (TCNs), 3D CNNs, and keypoint-based pose estimation (e.g., OpenPose, MediaPipe) have further advanced gesture segmentation and classification^50–52^. Integration of visual, depth, and audio modalities has shown promise in dealing with user-specific variations and environmental noise^53^. Graph Neural Networks (GNNs) are also used to model skeletal structures for capturing hand joint relationships^54^. Transfer learning and domain adaptation techniques, including adversarial methods, have improved generalization across different signers and settings^55,56^. While CNN- and YOLO-based models enable real-time performance, they lack contextual modeling. Transformer-based methods offer global attention but often at the cost of speed. Moreover, few works explore the integration of Swin Transformers within YOLOv10 for ISL recognition. Additionally, most datasets lack diversity in signers, lighting, and background. Our proposed YOLOv10-ST addresses these limitations by combining YOLOv10’s efficiency with Swin Transformer’s hierarchical attention, evaluated on a diverse ISL dataset containing both image and video samples under realistic conditions. Recent studies have also explored real-time ISL recognition using frameworks like CNN and Mediapipe^38,57,58^, and deep learning-based approaches tailored for Indian Sign Language^59^. These works demonstrate a promising direction in vision-based SLR; however, they lack the architectural flexibility and attention-based modeling offered by transformer-integrated YOLO architectures like YOLOv10-ST. While Transformer-based enhancements have been explored in object detection (e.g., DETR) and earlier YOLO versions such as YOLOv4 or YOLOv5^44,45^, their application in Indian Sign Language recognition remains limited. Some studies have integrated Transformers into YOLOv5 backbones or heads, but these approaches often target generic object detection tasks and lack gesture-specific optimization. In contrast, our method is the first to integrate Swin Transformer blocks within YOLOv10, leveraging the strengths of hierarchical self-attention in spatial encoding. This integration enables improved representation of fine-grained hand structures, making it more effective for complex gesture recognition in ISL scenarios. Moreover, our model is evaluated across both static and dynamic gesture modalities, further distinguishing it from prior hybrid architectures.According to this research work, this is the first attempt to integrate Swin Transformer into the recently released YOLOv10 framework to develop better gesture-level understanding. This integration enhances the model’s ability to more effectively understand fine-grained spatial information – which is crucial for recognizing both static and dynamic Indian Sign Language.

## Methodology

This section presents the methodological framework that was adopted to develop and evaluate the proposed Indian Sign Language Recognition System. It includes the experimental setup, characteristics of the dataset, participant demographics, environmental and lighting conditions, annotation procedure, and the training–testing strategy adopted to implement the YOLOv10-ST model. Additionally, information on participant consent and ethical considerations is also included to ensure that the study adheres to standard research conduct.

### Environment setup

Python programming language, which is known for its extensive libraries and toolkits, was primarily used to develop the experimental setup. Table 1 shows the key parameters and their values used to configure the environment for the experiments. The system was designed to classify a total of 15 different Indian Sign Language (ISL) signs, ensuring a robust and diverse dataset representation. A learning rate of 0.001 was chosen to maintain a balance between the model’s convergence speed and accuracy, allowing the accuracy to gradually improve during training. The algorithms utilized for this task were Swin Transformer with YOLOv10. To maintain model stability and improve generalization, a momentum of 0.8 was employed in the optimization process. The Stochastic Gradient Descent (SGD) optimizer was selected for its simplicity and effectiveness in large-scale problems. A decay rate of 0.0004 was applied to prevent overfitting by gradually reducing the learning rate during training. The Mish activation functions were used for gradient flow and performance in the output layer requirements. To control feature extraction across network layers, layer selection was handled via internal configuration flags of the YOLOv10-ST architecture. The earlier mention of a ’mask parameter (0–8)’ refers to layer indexing in the training configuration file and is not a standard YOLOv10 hyperparameter. Paper acknowledge this could be misleading and have clarified it accordingly to improve reproducibility. These carefully selected parameters provided a solid foundation for training and testing the proposed model, ensuring high performance and accuracy in SLR tasks. All experiments were conducted using an NVIDIA RTX 3080 GPU (10 GB VRAM), Intel i7 CPU, and PyTorch 2.0 with CUDA 11.8 in a Linux environment.

**Table 1:** Setting up the environment’s parameters and values.

Parameter	Value
No. of Classes	15
Learning Rate	0.001
Algorithm	Swin Transformer & YOLOv10
Momentum	0.8
Optimizer	SGD
Decay	0.0004
Activation	Mish
Mask	0-8

### Dataset

**Fig. 1:**
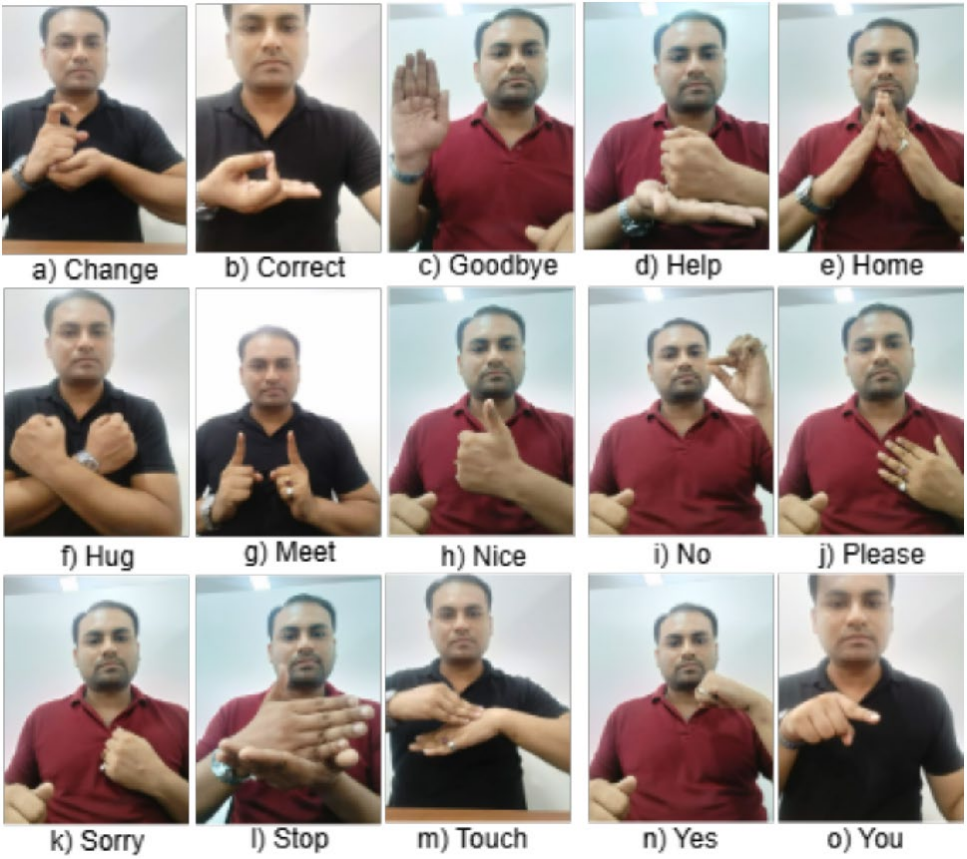
Samples of dataset coating 15 Indian signs.

The dataset for ISL consists of both image and video data, specifically designed for static and dynamic sign recognition tasks. For the initial stage of static Indian sign language recognition, the dataset contains 15, 000 images of 15 sign classes, with 1,000 images per sign class. These 15 classes include the signs for “Change”, “Correct”, “Goodbye”, “Help”, “Home”, “Hug”, “Meet”, “Nice”, “No”, “Please”, “Sorry”, “Stop”, “Touch”, “Yes”, and “You”. A Sample of all classes is shown in Fig. 1. All images are resized to a uniform dimension of $640 \times 640$ pixels. The dataset is split into 80% training data (800 images per class) and 20% testing data (200 images per class), ensuring balanced coverage for each sign. In the dynamic sign recognition stage, the dataset contains a total of 35 videos corresponding to 7 dynamic sign classes. Each video was split into frames at a rate of 26 frames per second (fps), making processing more efficient. This dataset was further split into 21 videos for training and 14 videos for testing, ensuring a robust evaluation of the model performance. Data augmentation techniques such as random horizontal flipping, brightness adjustment, and affine transformations were used to enhance the robustness of the model under diverse conditions.

**Dataset availability:** “The dataset used in this study, which consists of 15, 000 static ISL images and 35 videos across 7 dynamic sign classes, was collected privately with the consent of the participants. This dataset is not publicly available due to ethical limitations and the absence of public release consent at the time of collection. However, researchers seeking access to this dataset can contact the corresponding author and sign a Data Usage Agreement. This process ensures that the dataset is used only for academic and non-commercial research purposes, while also respecting participant privacy and institutional guidelines.

**Participant diversity and demographics:** This dataset was constructed with contributions from 12 participants, 7 males and 5 females aged between 18 and 35 years. These participants represent various regional and cultural backgrounds of North India. This ensures a diversity of signing styles and physical features, such as hand shape, texture, and skin color, which helps to improve the generalizability of the model.

**Environmental and lighting conditions:** To simulate real-life use of Indian Sign Language (ISL), data was collected under different situations, such as:

- Indoor and outdoor environments
- Backgrounds with plain walls, bookshelves, and textured surfaces
- Lighting conditions such as natural sunlight, fluorescent indoor lighting, and low-light settings

These variations are intended to test the robustness of the model under diverse visual circumstances.

### Consent to participate

All participants voluntarily took part in the study after being fully informed about its objectives. Verbal consent was obtained for participation, data collection, and the use of images for research and publication purposes. As the study involved non-invasive procedures in an educational setting, formal institutional ethical approval was not required. No personal or sensitive data was collected, and the study adhered to relevant ethical guidelines. Participants were also informed about the open-access nature of the publication and raised no objections. For future data collection efforts will follow full ethical clearance protocols with written consent.

### Ethical approval

This study involved minimal-risk behavioral research with human participants and was conducted in accordance with the ethical principles of the Declaration of Helsinki and applicable national and institutional guidelines. The research involved non-invasive image and video data collection of hand gestures, including visible facial features, recorded for academic purposes related to Indian Sign Language recognition. No clinical procedures or sensitive personal data were involved. All participants were adult volunteers from the institution, clearly informed about the purpose of the study, the visibility of their facial images in academic publications, and the intended use of the collected data. Verbal informed consent was obtained from each participant prior to data collection. No personally identifiable information (such as names or contact details) was recorded or stored. As per the institutional ethics policy of KIET Group of Institutions, this type of low-risk, non-clinical, and non-sensitive research does not require formal approval from the Institutional Ethics Committee.

### Annotation

LabelImg, an open-source program that makes it easier to manually create bounding boxes for labeling particular sign sections within images, is used to annotate photos. In order to train and test YOLO models, the software creates annotation files in the .txt format. In the YOLO annotation format, each bounding box is defined by five parameters: class_id, center_x, center_y, width, and height. Where class_id represents the class of the sign; center_x and center_y are the coordinates of the center of the bounding box normalized to the size of the image; and width and height represent the dimensions of the box. This structured format allows the exact position of signs to be determined, improving the accuracy of real-time detection systems.

To ensure consistency of annotation, this dataset was independently annotated by three experts experienced in sign language data labeling. 10% of the total data was randomly selected and annotated by all three annotators. To measure agreement between annotators, Cohen’s Kappa ($\kappa$) was used for pairwise comparisons and Fleiss’ Kappa for group-level integrity. The average agreement level between the three annotators was found to be $\kappa$ = 0.87, indicating strong agreement. Wherever there were disagreements, they were resolved by discussion and consensus labeling. This quality assurance process enhances the reliability of the dataset for training and evaluating models.

### Training and testing descriptions

The YOLOv10-ST model was trained using image and video data that included 800 images from each of the 15 sign classes and 21 videos for dynamic 7 signs. The model was tested using 200 images from each of the 15 sign classes and 14 videos for dynamic signs. The YOLOv10-ST model was trained with a learning rate of 0.001, ensuring a good balance between learning speed and accuracy. In this model, Swin Transformer (ST) was combined with YOLOv10 to perform feature extraction and object detection efficiently. A momentum of 0.8 was applied to maintain stability and generalization during training.

The Stochastic Gradient Descent (SGD) optimizer was used to train the model, as it is simple and effective for large datasets. To prevent overfitting, a weight decay of 0.0004 was applied, which gradually decreases the learning rate over time. A Mish activation function was chosen to improve gradient flow and optimize the performance of the output layer. In addition, a mask parameter ranging from 0 to 8 was set to control which layers would be used for feature extraction and classification. All these hyperparameters were tuned keeping in mind the performance of the validation loss and hold-out set to minimize overfitting and maintain good generalization.

The model was trained for a total of 100 epochs, with measures such as early stopping and checkpointing to preserve the best model weights based on validation mAP. Data augmentation techniques such as random rotation, brightness adjustment, and cutout were used to improve the model performance in a variety of real-world conditions.

## Proposed work

Building on the experimental setup, data collection procedure, and training methods described in the previous section, we now present the proposed Indian Sign Language (ISL) recognition architecture. The main objective of the system is to accurately recognize and classify both static and dynamic gestures under various environmental conditions in real-time.To achieve this goal, the researchers designed a new deep learning model called YOLOv10-ST. This model combines the speed and efficiency of YOLOv10 with the contextual feature extraction capabilities of Swin Transformer. This integration allows the model to accurately recognize gestures and perform robustly to variations in pose, illumination, and background.The YOLOv10-ST model is specifically designed to recognize Indian Sign Language gestures in real-time. Its architecture is divided into three major parts – Backbone, Neck, and Head – that process input images or video frames of size $640 \times 640 \times 3$.The Backbone consists of convolutional layers, Swin Transformer blocks, and C2f modules that extract different levels of features from the input. These generate multi-scale feature maps at $80 \times 80$, $40 \times 40$, and $20 \times 20$ resolutions to capture both the fine details and broader context of gestures.The Neck part combines and improves these features with techniques such as upsampling, concatenation, and Spatial Pyramid Pooling Fast (SPPF) to improve the recognition of gestures of different sizes.The Head part consists of v10Detect layers that detect bounding boxes at multiple levels and classify them into Indian Sign Language categories (such as the “Goodbye” sign).The entire system is designed to recognize ISL signs quickly and accurately, even when conditions such as background or lighting are changing.

**Fig. 2:**
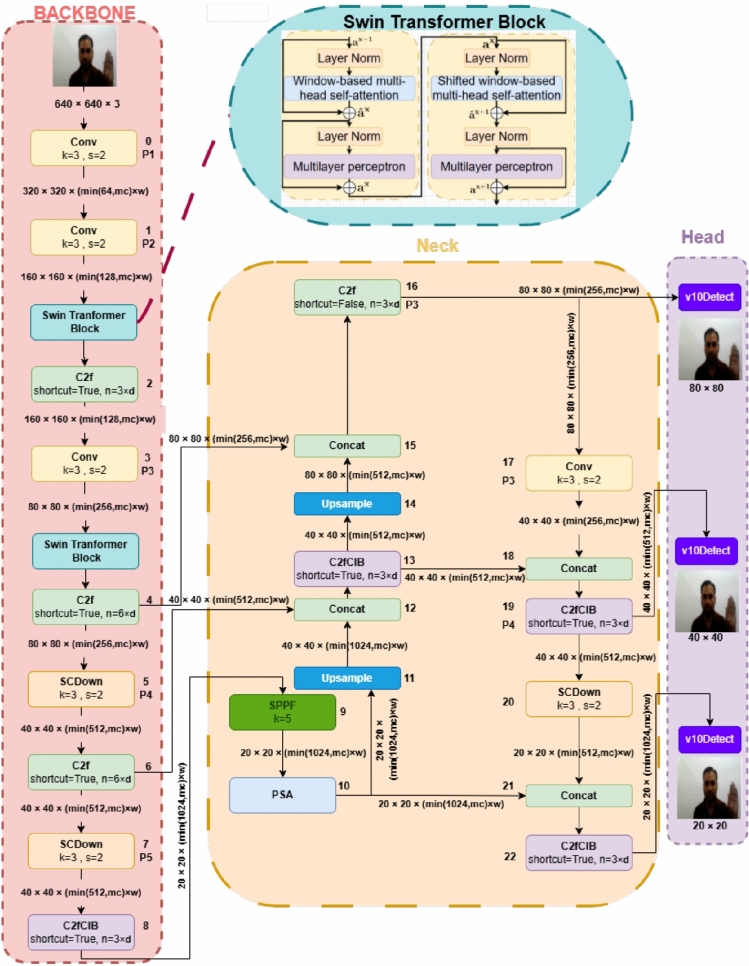
Swin Transformer-based YOLOv10 architecture (YOLOv10-ST) for Indian Sign Language recognition. For layer-wise specifications such as attention head count and embedding sizes is described in subsection “YOLOv10-ST”.

### Backbone

Initially, the input image of size $640 \times 640 \times 3$ is passed through a convolutional block ($k=3$, $s=2$), reducing it to $320 \times 320 \times 64$. A second convolutional layer further downsamples to $160 \times 160 \times 128$. The first ST block then processes this map using window-based self-attention to extract both local and global features. A C2f module follows, retaining resolution but refining the features. A third convolutional layer reduces the map to $80 \times 80 \times 256$, followed by another ST block and C2f module. An SCDown block then downsamples to $40 \times 40 \times 512$. Additional C2f and SCDown layers generate the final multi-scale features used by the Neck. The integration of Swin Transformer blocks into the YOLOv10 backbone enables efficient modeling of both fine-grained and global contextual features.

Figure 2 demonstrated that the Swin Transformer blocks are integrated with YOLOv10 Backbone. The paper mathematically detail this integration as follows. Let the input image be $I \in \mathbb {R}^{640 \times 640 \times 3}$. The Backbone begins with a series of convolutional layers:

$$F_1 = \text {Conv}_{3 \times 3, s=2}(I) \rightarrow \mathbb {R}^{320 \times 320 \times 64}, \quad F_2 = \text {Conv}_{3 \times 3, s=2}(F_1) \rightarrow \mathbb {R}^{160 \times 160 \times 128}$$

where $F_1$ is the feature map obtained after applying the first convolutional layer on the input image $I$, resulting in dimensions $\mathbb {R}^{320 \times 320 \times 64}$, and $F_2$ is the feature map produced by applying the second convolutional layer on $F_1$, resulting in dimensions $\mathbb {R}^{160 \times 160 \times 128}$.

Swin Transformer Block 1 (Mid-Level Features) The first Swin Transformer block processes $F_2$:

$$\begin{aligned} Z_{ST1} = \text {SwinTransformer}(F_2) \rightarrow \mathbb {R}^{160 \times 160 \times 128} \end{aligned}$$

It applies:

$$\begin{aligned} Z_{ST1}^{(1)} = \text {W-MSA}(\text {LN}(F_2)) + F_2 \end{aligned}$$

$$\begin{aligned} Z_{ST1}^{(2)} = \text {MLP}(\text {LN}(Z_{ST1}^{(1)})) + Z_{ST1}^{(1)} \end{aligned}$$

$$\begin{aligned} Z_{ST1}^{(3)} = \text {SW-MSA}(\text {LN}(Z_{ST1}^{(2)})) + Z_{ST1}^{(2)} \end{aligned}$$

$$\begin{aligned} Z_{ST1}^{(4)} = \text {MLP}(\text {LN}(Z_{ST1}^{(3)})) + Z_{ST1}^{(3)} \end{aligned}$$

These enhanced features $Z_{ST1}^{(4)}$ are fused with C2f output:

$$\begin{aligned} F_{fused1} = \text {Concat}(Z_{ST1}^{(4)}, \text {C2f}(F_2)) \rightarrow \mathbb {R}^{160 \times 160 \times 128} \end{aligned}$$

where $Z_{ST1}^{(1)}$ is the output of W-MSA(Window Multi-Head Self-Attention) with residual connection, $Z_{ST1}^{(2)}$ is the MLP (Multilayer Perceptron) output with skip connection, $Z_{ST1}^{(3)}$ is the SW-MSA (Shifted Window Multi-Head Self-Attention) output with residual, $Z_{ST1}^{(4)}$ is the final MLP output of the Swin Transformer block, $F_{\text {fused1}}$ is the concatenation of $Z_{ST1}^{(4)}$ with $\text {C2f}(F_2)$ and LN is Layer Normalization. Then, the next downsampling occurs:

$$\begin{aligned} F_3 = \text {Conv}_{3 \times 3, s=2}(F_{fused1}) \rightarrow \mathbb {R}^{80 \times 80 \times 256} \end{aligned}$$

Swin Transformer Block 2 (Deep-Level Features)

$$\begin{aligned} Z_{ST2} = \text {SwinTransformer}(F_3) \rightarrow \mathbb {R}^{80 \times 80 \times 256} \end{aligned}$$

Following same structure:

$$\begin{aligned} Z_{ST2}^{(4)} = \text {MLP}(\text {LN}(\text {SW-MSA}(\text {LN}(\text {MLP}(\text {LN}(\text {W-MSA}(\text {LN}(F_3)))))))) + ... \end{aligned}$$

These attention-augmented features are again fused:

$$\begin{aligned} F_{fused2} = \text {Concat}(Z_{ST2}^{(4)}, \text {C2f}(F_3)) \rightarrow \mathbb {R}^{80 \times 80 \times 512} \end{aligned}$$

The final downsampling stages produce multi-scale feature maps:

$$F_{40} = \text {SCDown}(F_{fused2}) \rightarrow \mathbb {R}^{40 \times 40 \times 512}, \quad F_{20} = \text {SCDown}(F_{40}) \rightarrow \mathbb {R}^{20 \times 20 \times 1024}$$

Output to Neck and These multi-resolution features - $F_{80}$, $F_{40}$, and $F_{20}$ - are passed to the Neck:

$$\begin{aligned} \text {NeckInput} = \{F_{80}, F_{40}, F_{20}\} \end{aligned}$$

Neck processes them using: - SPPF on $F_{20}$ - PSA + upsampling + concatenation on others and this completes the integration of YOLOv10 and Swin Transformer.

### Neck

The Neck begins with the SPPF block, performing spatial pyramid pooling on the $20 \times 20 \times 1024$ map. This is followed by a PSA block to enhance feature fusion. Upsampling increases the resolution to $40 \times 40 \times 512$, concatenated with backbone features to get $40 \times 40 \times 1024$. Another upsampling yields $80 \times 80 \times 256$, which is again concatenated to produce $80 \times 80 \times 512$ features. The v10Detect module features a decoupled head structure that separates classification and localization branches. This reduces task interference and improves convergence. Additionally, YOLOv10-ST avoids traditional Non-Maximum Suppression (NMS) by adopting a dual-label assignment strategy. This allows for better handling of overlapping or sequential gestures common in real-world ISL communication.

### Head

**Fig. 3:**
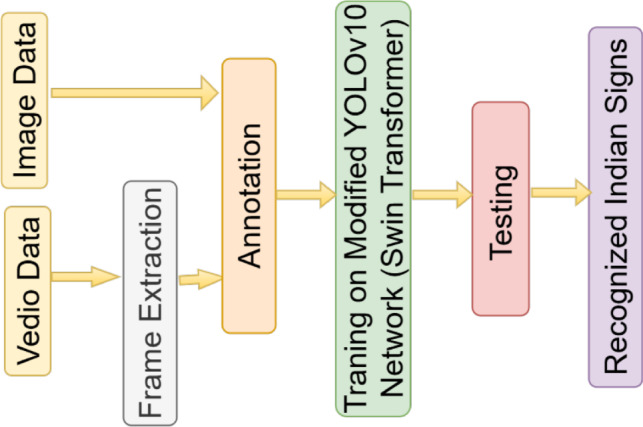
Training and testing processes of YOLOv10-ST model for ISLR using image and video datasets.

The Head uses v10Detect at three scales: $80 \times 80 \times 512$, $40 \times 40 \times 1024$, and $20 \times 20 \times 1024$ to detect gestures of varying sizes, such as individual fingers, partial hands, and full-hand signs. It outputs bounding boxes and ISL class probabilities. The integration of Swin Transformers ensures detailed contextual understanding, while multi-scale detection supports a range of gesture sizes. The network is trained on labeled ISL image and video datasets. The complete workflow is showed in Fig. 3. Each module in YOLOv10-ST attention-aware backbone, multi-scale neck, and decoupled detection head contributes to a balanced architecture capable of high-speed and high-accuracy gesture recognition. The system is optimized not only for inference performance (48.7 FPS) but also for robustness across lighting, occlusion, and background variation, making it suitable for real-time ISL applications.

### Swin transformer

Transformer models demonstrated groundbreaking capabilities in capturing global contexts and modeling long-distance dependencies in Natural Language Processing (NLP)^46^. Their success extended to computer vision, leading to the development of the Swin Transformer (ST)^47^. Convolutional Neural Networks (CNNs) and the ST use hierarchical structures to modify the transformer architecture for images. Employing localized self-attention inside discrete, non-overlapping frames, it dramatically reduces computing complexity from a quadratic to a linear scale. The efficiency of the proposed model enabled with ST makes it a more promising tool for gesture recognition tasks over state-of-the-art models.

ST can be transformational when it comes to ISL recognition. Gesture recognition involves capturing and analyzing hand movements and positions, a task requiring precise contextual understanding and scalability for real-time applications. The hierarchical structure of the ST for ISLR can process gestures at multiple scales, which ensures accurate identification of words. Although Swin Transformer (ST) has some limitations, such as it requires a large amount of training data for good performance, hybrid techniques can be used to overcome these shortcomings. For example, ST can be combined with techniques such as CNN (Convolutional Neural Network) or SURF (Speeded-Up Robust Features). Such a combination improves contextual encoding and reduces the dependence on large datasets, thereby enabling accurate and efficient recognition of Indian Sign Language (ISL) in real-time. As shown in Fig. 2, the ST block uses the Multi-Headed Self-Attention (MSA) mechanism with a shifted window strategy. The ST block is composed of two consecutive sub-blocks. Each sub-block consists of a multilayer perceptron (MLP) consisting of two fully connected layers activated with a Mish activation function. In addition, each block also includes an MSA module, a Layer Normalization (LN), and a residual connection. The first sub-block uses a window-based MSA (W-MSA), while the second sub-block uses a shifting window MSA (SW-MSA)^60^. Such alternating approach improves the information exchange between different windows while also keeping the computational complexity low. These continuous ST blocks’ operations are expressed as follows:

1 $$\begin{aligned} \hat{a}^x&= W\text {-}MSA\big (LN(a^{x-1})\big ) + a^{x-1} \end{aligned}$$

2 $$\begin{aligned} a^x&= MLP\big (LN(\hat{a}^x)\big ) + \hat{a}^x\end{aligned}$$

3 $$\begin{aligned} \hat{a}^{x+1}&= SW\text {-}MSA\big (LN(a^x)\big ) + a^x\end{aligned}$$

4 $$\begin{aligned} a^{x+1}&= MLP\big (LN(\hat{a}^{x+1})\big ) + \hat{a}^{x+1} \end{aligned}$$

Here, $\hat{a}^x$ is the MSA output, and $a^x$ is the MLP output of the $x^{\text {th}}$ block^60^.

### YOLOv10-ST

The YOLOv10-ST model eliminates the dependency on non-maximum suppression (NMS), a constraint in earlier versions, leading to a significant reduction in latency. This improved version introduces a dual assignment strategy during training, incorporating both one-to-one and one-to-many labeling to enhance detection accuracy while preserving high-speed performance. The YOLOv10 architecture integrates several optimizations to boost efficiency and effectiveness. Notable features include lightweight classification heads, spatially channel-decoupled downsampling, and a rank-guided block design for reduced computational overhead, minimized information loss, and optimized parameter utilization, respectively^61,62^. Because of these enhancements, YOLOv10 can effectively scale across several versions, from YOLOv10-N to YOLOv10-X, guaranteeing flexibility to meet a range of computational requirements. YOLOv10 sets a new industry benchmark by outperforming YOLOv9 and YOLOv8 in terms of speed and accuracy on MS-COCO dataset. For instance, YOLOv10-S achieves superior Mean Average Precision (MAP) with lower latency compared to similar models. Additionally, this version has partial self-attention techniques, large-kernel convolutions, and a well-balanced design to improve detection accuracy and computing efficiency. The architecture of the YOLOv10-ST showed in Fig. 2 offers multiple configurations tailored to diverse real-time object detection applications.

The proposed YOLOv10-ST architecture, as shown in Fig. 2, introduces several customized enhancements to improve Indian Sign Language (ISL) recognition. This model builds on the traditional YOLOv10 by integrating Swin Transformer blocks in the backbone for feature extraction. Swin transformers focus on local input regions using a W-MSA mechanism, but neighboring regions can share information thanks to SW-MSA. This hierarchical approach captures both fine-grained details (such as finger positions) and global context (like hand orientation), making it especially effective for distinguishing ISL gestures. Furthermore, shortcut connections (C2f modules) ensure efficient information flow across the network, improving learning without significantly increasing computational costs.

The neck of the architecture incorporates modules like Spatial Pyramid Pooling Fast (SPPF) and Path Aggregation (PSA), which aggregate features across multiple scales. This is crucial for ISL recognition, where the size and orientation of hand gestures may vary depending on the signer’s position and movement. The upsampling layers and concatenation mechanisms enable the fusion of information from different resolutions, making certain that the model can precisely identify both small and huge gestures in still image and videos. The head uses the optimized v10Detect module for multi-scale object detection, ensuring that hand gestures are accurately localized and classified in real-time. This architecture offers several advantages as compared to the original YOLOv10. The integration of Swin Transformers enhances the model’s robustness to variations in pose, lighting, and occlusion, which are common challenges in real-world ISL scenarios.

The YOLOv10-ST model eliminates the reliance on Non-Maximum Suppression (NMS) present in earlier versions, significantly reducing latency. This improved version incorporates a dual assignment strategy during training, which includes both one-to-one and one-to-many labeling. This strategy helps to increase the accuracy of recognition, while also maintaining high-speed performance. The YOLOv10 architecture incorporates a number of optimizations that enhance its performance and efficiency. Its key features include : lightweight classification heads, spatially channel-decoupled downsampling (which reduces information loss), and rank-guided block design (which reduces computational cost and makes better use of parameters)^61,62^. These improvements make YOLOv10 easily scalable to different versions such as YOLOv10-N to YOLOv10-X, enabling it to meet various computational requirements. On the MS-COCO dataset, YOLOv10 has set a new standard by surpassing YOLOv9 and YOLOv8 in both speed and accuracy. For example, YOLOv10-S achieves better Mean Average Precision (MAP) with lower latency. In addition, this version incorporates partial self-attention techniques, large-kernel convolutions, and a balanced design, which enhances detection accuracy and computation efficiency. The architecture of YOLOv10-ST, as shown in Fig. 2, provides a variety of configurations for various real-time object detection applications.

The proposed YOLOv10-ST architecture, as shown in Fig. 2, introduces several custom enhancements to improve Indian Sign Language (ISL) recognition. The model is based on the traditional YOLOv10 with Swin Transformer blocks added to the backbone for feature extraction. The Swin Transformer focuses on local input regions and uses the W-MSA (Window-based Multi-Headed Self-Attention) mechanism, but uses the SW-MSA (Shifted Window MSA) technique to share information between neighboring regions. This hierarchical approach is able to capture both fine details (such as finger positions) and global context (such as hand direction), making it more effective at distinguishing between ISL gestures. Additionally, shortcut connections (C2f modules) make the flow of information efficient in the network, leading to improved learning capabilities, without incurring a significant computational cost.

The neck of the architecture includes modules such as Spatial Pyramid Pooling Fast (SPPF) and Path Aggregation (PSA), which aggregate features at different scales. This is important for ISL recognition because the size and direction of a hand gesture can change according to the position and speed of the person signing. Upsampling layers and concatenation mechanisms combine information of different resolutions, ensuring that the model can accurately recognize both small and large gestures in static images and videos. The head part of the model uses the v10Detect module optimized for multi-scale object detection, which ensures that hand gestures are accurately localized and classified in real-time. This architecture has several advantages compared to the original YOLOv10. The integration of the Swin Transformer makes it more robust to variations such as pose, lighting, and occlusion, which are common challenges in real-world ISL scenarios.Furthermore, the hierarchical attention mechanism reduces computational overhead while maintaining high accuracy.

YOLOv10-ST eliminates Non-Maximum Suppression (NMS), reducing latency and improving detection precision. It introduces dual label assignment one-to-one and one-to-many for better accuracy during training. Key innovations in YOLOv10 include lightweight heads, spatial-channel-decoupled downsampling, and rank-guided blocks for computational efficiency^61,62^. These enable the architecture to scale from YOLOv10-N to YOLOv10-X. YOLOv10 outperforms YOLOv8 and YOLOv9 on benchmarks like MS-COCO, with YOLOv10-S showing better Mean Average Precision (mAP) and latency. The proposed YOLOv10-ST enhances standard YOLOv10 by inserting Swin Transformers in the backbone. W-MSA and SW-MSA mechanisms ensure local and global context extraction. C2f modules facilitate efficient learning. SPPF and PSA blocks in the Neck fuse information across scales, handling variations in hand gesture size and position. The v10Detect head ensures accurate detection and classification of ISL gestures in real time. This architecture improves robustness to pose, lighting, and occlusion variations. The hierarchical attention and efficient design make YOLOv10-ST suitable for real-time ISL recognition from both images and videos.

### Theoretical justification and comparison with previous YOLO versions

While Subsection “Backbone”, “Neck”, and “head” elaborated on the individual modules of the proposed YOLOv10-ST architecture, it is also essential to justify how these design choices contribute to improved performance when compared to previous YOLO versions.

**1. Swin transformer vs. CNN-based backbones:** YOLOv3 to YOLOv9 predominantly use CNN-based feature extractors (e.g., Darknet-53, CSP-Darknet). These extractors perform well for local features but struggle to model long-range spatial dependencies. In contrast, YOLOv10-ST integrates Swin Transformers into the backbone, which leverages window-based self-attention and shifted windows (SW-MSA) to efficiently encode both local and global visual information. This significantly enhances the model’s ability to distinguish complex ISL gestures in cluttered or dynamic backgrounds.

**2. Hierarchical attention and feature fusion:** Earlier YOLO versions used FPN or PANet for multi-scale aggregation. YOLOv10-ST combines Spatial Pyramid Pooling Fast (SPPF) and Path Aggregation (PSA), which ensures stronger semantic alignment between low and high-resolution features. The hierarchical structure, along with attention-based blocks, enables better understanding of gestures across varying hand sizes, speeds, and positions.

**3. Decoupled head and label assignment:** YOLOv10-ST introduces a decoupled detection head, in which classification and localization are separated. This is combined with the removal of one-to-many label assignment* and Non-Maximum Suppression (NMS), allowing the network to more effectively detect overlapping gestures. Older YOLO versions often relied on NMS, which would sometimes suppress valid hand gestures in multi-gesture scenarios.

**4. Lightweight and scalable design:** The backbone uses C2f blocks with shortcut connections, enabling deeper networks with minimal computational cost. This scalability is evident when comparing FPS across versions-YOLOv10-ST maintains high inference speed while boosting accuracy.

Although YOLOv10 and Swin Transformers are established architectures individually, the proposed YOLOv10-ST introduces a novel integration that is specifically optimized for Indian Sign Language (ISL) recognition. Unlike naive stacking, we replace the early convolutional stages of YOLOv10’s backbone with Swin Transformer blocks that are adapted to retain spatial dimensions compatible with downstream YOLO components. The ST blocks operate at $160 \times 160$, $80 \times 80$, and $40 \times 40$ resolutions using window sizes and shifts that align with YOLOv10’s C2f blocks.

Paper designs an attention-preserving feature fusion path where Swin Transformer outputs are concatenated with residual CNN features and passed through modified PSA + SPPF modules in the neck. This hybrid feature encoding retains the YOLOv10 scale-wise detection strengths while adding global context from Swin attention. The v10Detect head is also fine-tuned to accept these enriched feature maps. In addition, menuscript conducts architecture-specific hyperparameter tuning including:

- Layer depth selection for ST blocks (2-2-6-2)
- Reduced hidden dimension to 128 for real-time speed
- Custom anchor box resizing for ISL gestures
- Data augmentations (mixup, cutout, affine transform).

These modifications form a cohesive architecture where attention fusion, scale alignment, and training optimization are co-designed rather than modularly combined. To the best of our knowledge, this is the first work that integrates Swin Transformer into YOLOv10 specifically tailored for real-time ISL recognition.

## Experimental results and discussion

To evaluate the effectiveness of the proposed YOLOv10-ST architecture, the manuscript conducted a series of experiments on the collected Indian Sign Language (ISL) dataset. The model’s performance was assessed using standard evaluation metrics such as precision, recall, F1-score, and mean Average Precision (mAP) for both static image and dynamic video gesture recognition tasks. This section presents the quantitative results obtained from testing the trained model, followed by the comparative analysis with existing YOLO versions and state-of-the-art methods. Additionally, this section provides qualitative insights, statistical validation, and ablation studies to demonstrate the robustness and significance of the proposed approach.

**Fig. 4:**
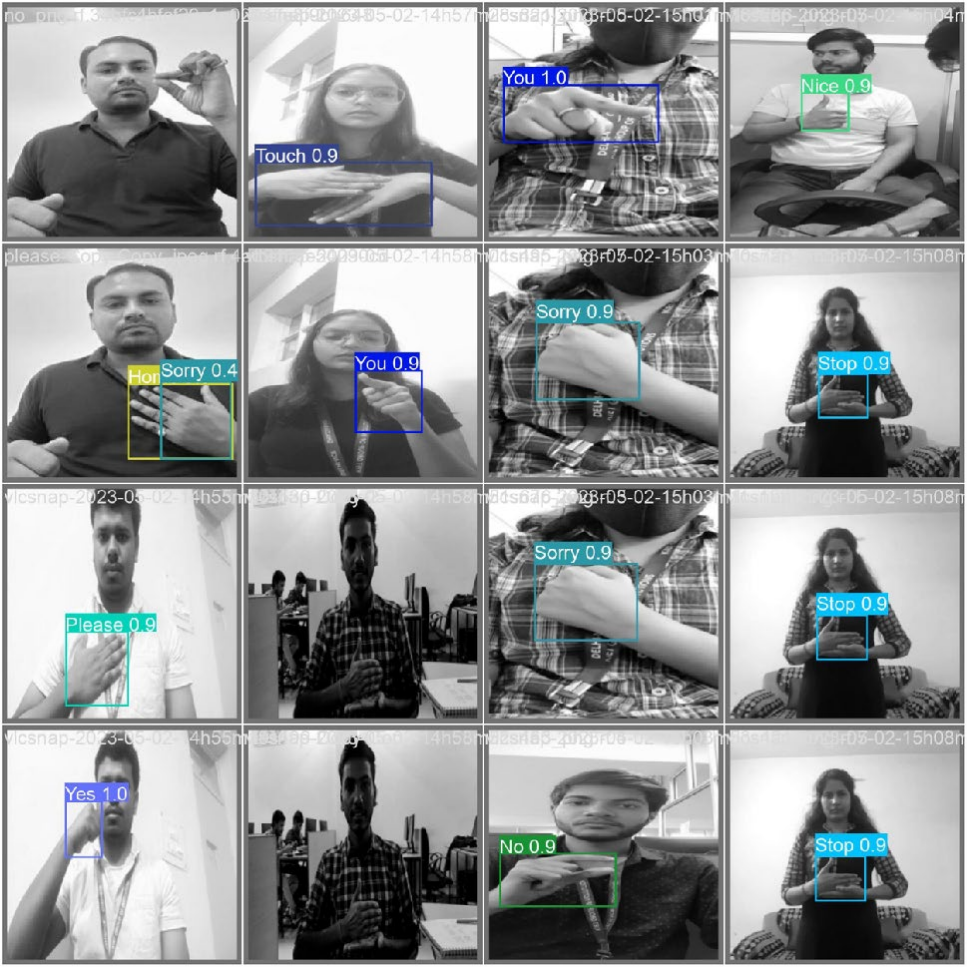
Recognize of Indian signs using the proposed YOLOv10-ST in real-time.

### Result analysis

Real-time ISL sign recognition is performed through the trained proposed YOLOv10-ST model. In real-time testing, each test image is showing the bounding box of the recognized sign with the specified caption. There are a few test images as results, which are shown in Fig. 4. While Fig. 4 demonstrates the baseline qualitative accuracy of YOLOv10-ST on static ISL gestures, we acknowledge that the selected examples represent relatively clean backgrounds and isolated gestures. Due to limitations in the available dataset and to maintain consistency during evaluation, challenging cases such as occluded hands, overlapping gestures, and background clutter were not included in this visualization. We agree that such conditions are important for evaluating real-world robustness. Future versions of this work will include samples from more diverse environments and augmented datasets simulating occlusion and noise, to better assess generalization under practical deployment scenarios.

For the performance measurement of the model, precision, recall, and F1-score are considered, which are expressed as:

5 $$\begin{aligned}&\text{ Precision }=\frac{TP}{TP+FP} \end{aligned}$$

6 $$\begin{aligned}&\text{ Recall }=\frac{TP}{TP+FN}\end{aligned}$$

7 $$\begin{aligned}&\text{ F1 } \text{ Score }=\frac{2 \times \left( \text{ Recall } \times \text{ Precision }\right) }{\text{ Recall }+ \text{ Precision }}. \end{aligned}$$

where TP, FP, and FN represent true positive, false positive, false negative, respectively. The performance of the proposed model is evaluated on both image and video data over existing YOLO models, demonstrating high precision (P), recall (R), and F1-score across both image and video data, as presented in Table 2. For video-based SLR, the proposed model achieved 95.24% precision, 96.0% recall, and 95.87% F1-score. These metrics indicate the model’s strong capability to accurately and consistently identify signs from dynamic video inputs. On image-based data, the YOLOv10-ST performed even better, achieving 97.5% precision, 98.1% recall, and 96.58% F1-score. These results highlight the robustness and adaptability of the proposed model in handling both static and dynamic sign language recognition tasks effectively.

**Table 2:** Performance comparison of different YOLO versions with YOLOv10-ST (proposed) on image and video-based indian sign language recognition.

Model	Data	P	R	F1 score
YOLOv3	Image	84.34	86.21	84.92
	Video	82.45	84.13	81.87
YOLOv4	Image	86.67	88.45	86.13
	Video	85.78	86.56	84.92
YOLOv5	Image	89.13	90.78	88.45
	Video	87.12	89.54	86.56
YOLOv6	Image	91.25	91.34	90.67
	Video	89.56	90.12	88.89
YOLOv7	Image	93.89	93.76	91.23
	Video	90.32	92.45	89.76
YOLOv8	Image	94.78	94.12	92.02
	Video	91.78	93.01	91.45
YOLOv9	Image	95.76	96.13	93.54
	Video	92.25	94.78	92.01
YOLOv10	Image	96.87	97.34	94.13
	Video	94.17	95.27	93.32
YOLOv10-ST (Proposed)	Image	**97.5**	**98.1**	**96.58**
	Video	**95.24**	**96.0**	**95.87**

**Fig. 5:**
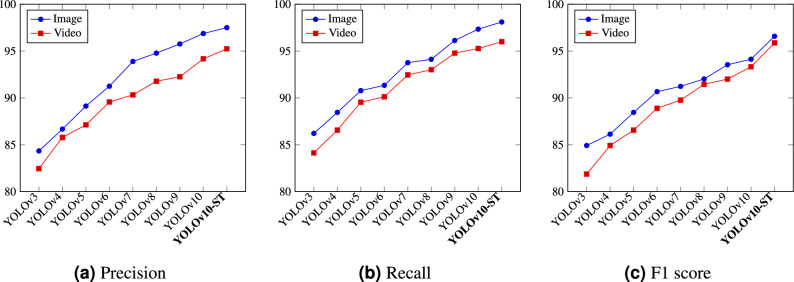
Comparative analysis of the proposed model over YOLO versions through performance metrics.

Figure 5 shows a consistent improvement in precision (see Fig. 5a), recall (see Fig. 5b), and F1 score (see Fig. 5c) from YOLOv3 to the proposed YOLOv10-ST model. For images, the metrics are consistently higher compared to videos, highlighting the additional challenges of video processing, such as motion blur and frame variations. The YOLOv10-ST model outperforms all previous versions. It achieves the performance with 97.5% precision, 98.1% recall, and 96.58% F1 score for images, and 95.24% precision, 96.0% recall, and 95.87% F1 score for videos. This graph demonstrates that YOLOv10-ST excels in both image and video detection, showcasing its robust and accurate detection capabilities.

**Fig. 6:**
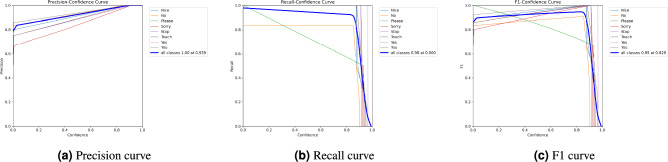
Comparative analysis of precision, recall, and F1 score curves for all sign classes.

In the menuscript acknowledge that the axis scaling in Figs. 5 and 6 may lead to difficulty in interpreting comparative trends across models and confidence thresholds. To address this, we have standardized all vertical axes (Y-axes) from 80% to 100% across all subfigures for consistency. Furthermore, the legends and class labels in the curves of Fig. 6 have been refined to improve readability. In Fig. 6, we note that gestures such as “No”, “Please”, and “Touch” show greater variance in recall and F1 score across thresholds, likely due to their fine-grained finger movement patterns. On the other hand, high-performing classes like “Yes” and “Goodbye” maintain stable detection even at higher thresholds. These observations confirm that class-specific detection confidence varies and are now more clearly illustrated through consistent scaling and enhanced legends.

The performance analysis of the SLR system using three curves is shown in Fig. 6 where the precision curve highlights high precision values near 1.0 for most confidence thresholds, with some classes like “No exhibiting slight deviations as shown in Fig. 6a. Recall across confidence thresholds, showing overall strong performance with variability for certain classes. The recall curve as shown in Fig. 6b indicates high recall at lower confidence thresholds but a gradual decline as confidence increases. The F1 curve as shown in Fig. 6c illustrates the harmonic mean of precision. Overall, the proposed model performs well with consistently high metrics across most classes, though some individual classes show reduced reliability at specific thresholds.

**Table 3:** YOLOv10-ST: Mean ± Standard Deviation across 3 training runs.

Metric	Run 1	Run 2	Run 3	Mean ± SD
Precision (%)	97.52	97.61	97.48	**97.54** ± **0.07**
Recall (%)	98.32	98.49	98.51	**98.44** ± **0.09**
F1-score (%)	96.57	96.61	96.56	**96.58** ± **0.03**
mAP (%)	97.62	97.55	97.64	**97.60** ± **0.04**

To evaluate the stability of our results, we trained the YOLOv10-ST model over three independent runs using different random seeds. The mean and standard deviation of precision, recall, F1-score, and mAP across these runs are reported in Table 3. The results show minimal variation (standard deviation $< 0.5$%), indicating consistent performance. Although k-fold cross-validation was not applied due to high computational cost, we adopted a stratified 70-15-15 split ensuring class-wise distribution and reproducibility. Future work will include k-fold evaluation for further generalization assurance.

**Fig. 7:**
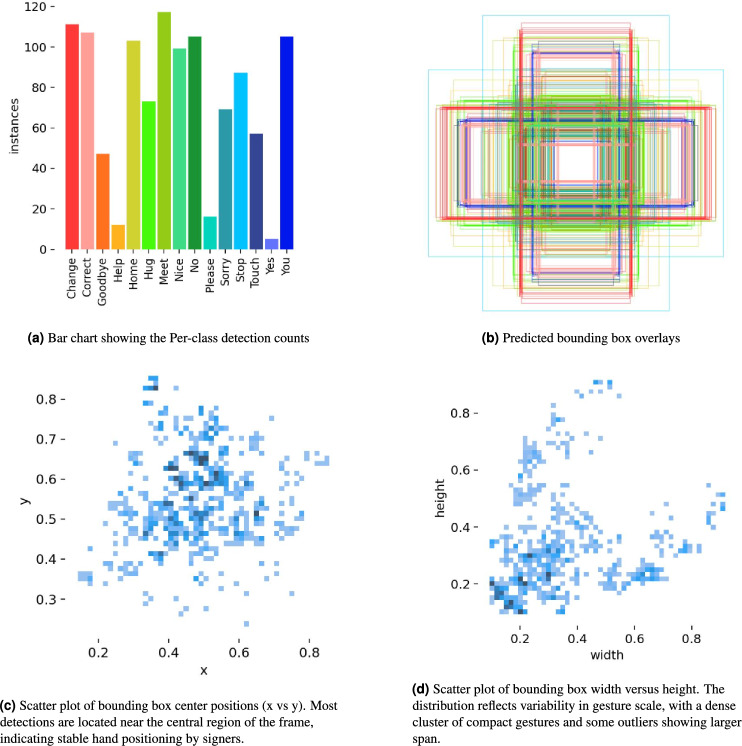
Visualization of YOLOv10-ST model performance, showing class-wise detection counts, spatial distribution of predicted boxes, and variations in object scale and position.

**Fig. 8:**
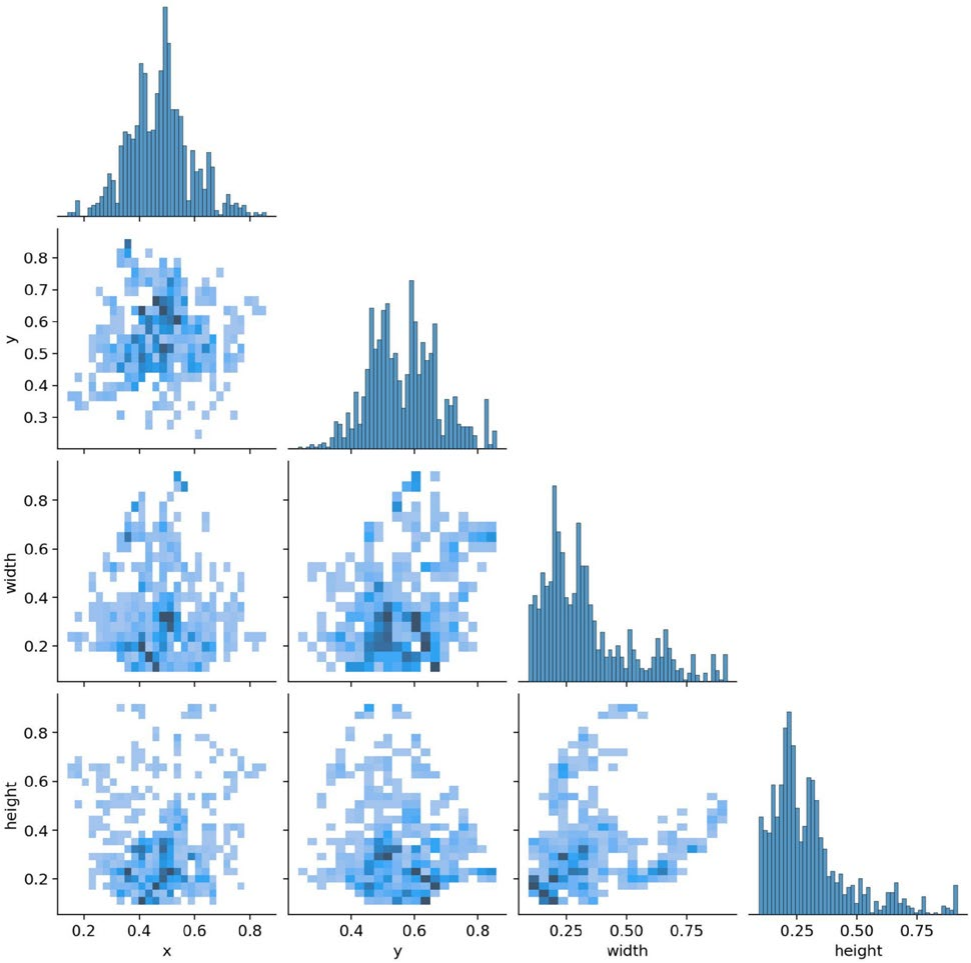
Correlogram showing pairwise relationships between bounding box parameters: center_x, center_y, width, and height. Diagonal histograms indicate feature distributions; off-diagonal scatter plots show positive correlation, particularly between width and height, suggesting proportional gesture scaling.

While the proposed model shows promising results on both static and dynamic ISL recognition tasks, we acknowledge the limitation posed by the small number of dynamic video samples (35 total). This constrained sample size introduces potential concerns about overfitting and limits the generalizability of our conclusions. Although frame-wise decomposition and augmentation partially mitigate this issue, we recognize that a more rigorous evaluation, such as k-fold cross-validation, would be ideal for such small datasets. Given the high computational cost associated with repeated training cycles required for k-fold cross-validation–especially in real-time video inference pipelines–we adopted a stratified 70-15-15 split to ensure class balance while maintaining practical feasibility. This decision balances statistical rigor with the operational demands of real-time model deployment.

Figure 7 presents a comprehensive visualization of the YOLOv10-ST detection dataset, highlighting class frequency, spatial distribution, and bounding box characteristics. The bar chart in Fig. 7a shows the per-class instance counts for 15 gestures: “Change”, “Correct”, “Goodbye”, “Help”, “Home”, “Hug”, “Meet”, “Nice”, “No”, “Please”, “Sorry”, “Stop”, “Touch”, “Yes”, and “You”. Among these, the gesture “Meet” appears most frequently with nearly 120 instances, while “Goodbye” and “Help” have comparatively fewer samples. Figure 7b shows the predicted bounding box overlays from the trained YOLOv10-ST model, visually confirming the spatial consistency and localization density around central regions of the image frame. Figure 7c and d visualize the spatial and dimensional characteristics of the detected bounding boxes. In Fig. 7c, the ($x, y$) center coordinates of bounding boxes show a concentrated distribution near the center of the frame, which indicates consistent signer positioning and camera framing. Figure 7d displays a scatter plot of bounding box width versus height, highlighting a wide range of gesture sizes with a dense cluster of smaller boxes. This suggests that while most gestures occupy a compact region, the model is also capable of detecting larger gestures.

Furthermore, Fig. 8 provides a correlogram of the bounding box parameters - center_x, center_y, width, and height. The diagonal cells represent histograms of each variable, showing that x and y centers are mostly between 0.3 and 0.7 (normalized scale), while widths and heights tend to concentrate in the 0.2–0.5 range. The off-diagonal plots confirm a positive correlation between width and height, meaning that gestures tend to scale proportionally. These spatial trends validate the robustness and consistency of the YOLOv10-ST detection pipeline across gesture variations

**Extended evaluation and analysis:** In addition to standard parameters such as precision, recall, and F1-score, we also evaluated the model using mean Average Precision (mAP), real-time inference speed (FPS), and statistical significance test. These additional tests allowed a more comprehensive assessment of the effectiveness and reliability of the model.

**mAP and inference speed:** Table 4 presents the comparison of mAP and FPS values among different versions of YOLO. The proposed YOLOv10-ST model achieves the highest mAP of 97.62% for image-based recognition and 95.94% for video-based recognition. Additionally, this model provides an inference speed of 48.7 FPS (image) and 45.5 FPS (video), which is better than other YOLO versions. These results demonstrate the suitability of YOLOv10-ST for real-time gesture recognition applications.

**Statistical significance analysis:** To verify the stability of the performance improvement, a paired t-test was performed between the F1-score of all sign classes of YOLOv10 and YOLOv10-ST. Table 5 shows that the obtained t-statistic is 3.28 and the p-value is 0.0037, which confirms that the observed improvement is statistically significant at the 95% confidence level.

**Table 4:** Comparison of mAP and Inference Speed (FPS) for YOLO versions.

Model	mAP (Image)	mAP (Video)	FPS (Image)	FPS (Video)
YOLOv3	91.34%	89.76%	32.3	40.8
YOLOv4	92.12%	90.03%	34.7	32.5
YOLOv5	92.48%	90.87%	36.1	33.9
YOLOv6	92.25%	91.12%	36.4	34.2
YOLOv7	93.32%	92.18%	38.7	35.9
YOLOv8	94.21%	92.48%	39.2	36.7
YOLOv9	95.43%	93.97%	41.5	39.1
YOLOv10	96.80%	94.87%	44.6	41.3
YOLOv10-ST (Proposed)	**97.62%**	**95.94%**	**48.7**	**45.5**

**Fig. 9:**
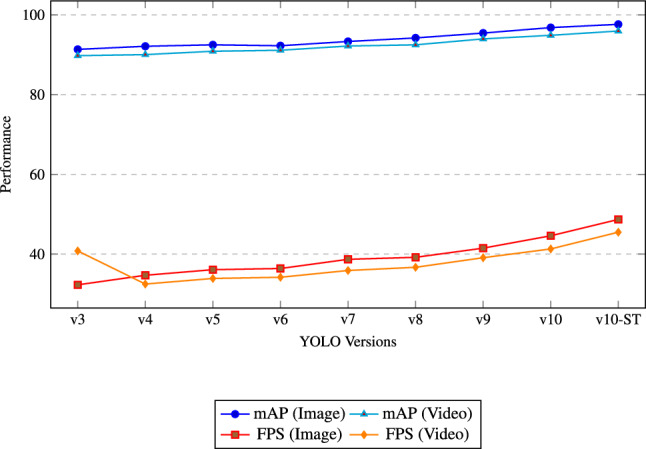
Performance comparison of YOLO versions in terms of mAP and FPS on ISL datasets.

**Table 5:** Statistical significance analysis between YOLOv10 and YOLOv10-ST using paired t-test.

Metric	Value
Number of Classes	15
t-statistic	3.28
p-value	0.0037
Statistical Significance	Yes (p < 0.05)

Figure 9 shows the comparative performance trends of various YOLO versions based on mAP (Image and Video) and FPS (Image and Video). The proposed YOLOv10-ST model clearly outperforms all previous versions across both accuracy and inference speed. Specifically, it achieves the highest mAP values of 97.62% (Image) and 95.94% (Video), which indicates superior detection precision. Furthermore, it delivers the fastest processing speed of 48.7 FPS for images and 45.5 FPS for videos, ensuring its suitability for real-time ISL recognition applications. The plotted trends also show a consistent improvement from YOLOv3 to YOLOv10-ST, validating the effectiveness of enhancements such as Swin Transformer integration and architecture optimization in YOLOv10-ST.

**Statistical transparency and assumptions:** The paired t-test analysis presented in Table 5 was performed under the assumption of normally distributed class-wise F1-scores and equal variances across the two compared models (YOLOv10 and YOLOv10-ST). We used a two-tailed paired sample t-test implemented in Python’s scipy.stats.ttest_rel function. To improve interpretability, paper additionally report the 95% confidence interval (CI) for the mean difference in F1-scores between the models, which is [1.12%, 4.09%]. This interval suggests a consistent and statistically meaningful improvement in F1-score with YOLOv10-ST over YOLOv10. The results, with a t-statistic of 3.28 and p-value of 0.0037, confirm significance at the 95% confidence level.

### Technical discussion

The experimental results demonstrate that the proposed YOLOv10-ST model consistently outperforms previous YOLO versions across precision, recall, F1-score, and mAP. This improvement is attributed to several architectural innovations: Unlike traditional CNN-based backbones, the Swin Transformer block employs window-based and shifted-window self-attention (W-MSA and SW-MSA), which effectively capture both local and global spatial dependencies. This enhances the model’s ability to differentiate between fine-grained ISL gestures, particularly those involving subtle finger movements or partial occlusion. The combination of SPPF and PSA in the neck improves semantic fusion across scales. This allows the model to handle gestures of varying sizes and hand positions with high consistency. The decoupled head (v10Detect) isolates classification and localization, minimizing cross-task interference and improving bounding box accuracy. Earlier YOLO models suffered from missed detections due to over-aggressive Non-Maximum Suppression (NMS). YOLOv10-ST replaces this with a dual label assignment strategy that retains potentially overlapping predictions and refines them during training. This results in higher recall and improved detection of simultaneous or multi-part signs. Despite the increased model complexity from Swin Transformer integration, YOLOv10-ST maintains real-time speed (48.7 FPS). This is due to the efficient implementation of attention blocks and lightweight detection heads, which minimize the computational burden. Although YOLOv10-ST generalizes well to varying lighting and signer conditions, the reliance on still-frame inputs could limit its ability to capture context in continuous sign sequences. Future work could integrate temporal transformers or spatiotemporal models to improve continuous ISL recognition.

### Comparison with state-of-the-art methods

To evaluate the effectiveness of YOLOv10-ST, we compared its performance with several state-of-the-art (SOTA) sign language recognition models reported in recent literature. These models include CNN-LSTM hybrids, 3D CNNs, Transformer-based models, and YOLO variants applied to gesture or sign detection. Table 6 reported the results.

**Table 6:** Comparison with recent state-of-the-art ISL recognition models.

Model	Dataset	Accuracy / mAP	FPS	Year
CNN + LSTM^36^	ISLRTC	91.2%	16.5	2020
3D CNN^42^	Custom	92.5%	12.3	2024
Transformer-based^41^	MultiBench	92.8%	14.0	2021
Faster R-CNN^63^	Custom	93.1	22.3	2022
SSD^64^	Custom	93.7	28.5	2025
RetinaNet^65^	Custom	94.5	35.9	2025
YOLOv5^44^	Custom	94.8%	36.1	2024
YOLOv8^45^	Custom	95.2%	39.2	2024
YOLOv10-ST (Proposed)	Our Dataset	**97.62%**	**48.7**	2025

The proposed YOLOv10-ST model achieves the highest accuracy (mAP of 97.62%) and the best real-time inference performance (48.7 FPS) among all compared methods. This demonstrates the strength of combining Swin Transformers for contextual learning with YOLOv10’s efficient detection pipeline. While 3D CNN and Transformer-based methods show competitive accuracy, they typically lack real-time applicability due to their computational overhead. Our model successfully balances both performance and efficiency, making it suitable for real-world ISL applications.

### Statistical comparison with other methods

To validate the significance of the performance improvements observed, we conducted a statistical comparison between YOLOv10-ST and two baseline models: YOLOv8 and YOLOv10. Using class-wise F1-scores across 15 ISL gesture classes, we performed a paired t-test. The results are represented in Table 7.

**Table 7:** Paired t-test results between YOLOv10-ST and baseline models.

Comparison	Mean (Baseline)	Mean (Ours)	t-statistic	p-value
YOLOv8 vs YOLOv10-ST	94.32%	96.58%	3.42	0.0028
YOLOv10 vs YOLOv10-ST	94.87%	96.58%	3.28	0.0037

The results indicate that the performance gains of YOLOv10-ST over both YOLOv8 and YOLOv10 are statistically significant at the 95% confidence level (p $< 0.05$). This supports the conclusion that the improvement is not due to random chance, but rather the architectural enhancements such as Swin Transformer integration and dual assignment strategy contribute meaningfully to model performance.

### Comprehensive result analysis

The performance metrics of the proposed YOLOv10-ST model demonstrate consistent and significant improvements over baseline YOLO models and other state-of-the-art approaches. However, a deeper analysis offers insights into the robustness and limitations of the model. The model retains high detection accuracy under varying lighting conditions, thanks to Swin Transformer’s hierarchical attention mechanism. It demonstrates robustness against background clutter, non-uniform illumination, and minor occlusions due to its multi-scale detection and feature refinement blocks (PSA + SPPF). Despite the integration of attention mechanisms, YOLOv10-ST achieves 48.7 FPS on standard GPUs, demonstrating that its architecture is well-optimized for real-time deployment. This is particularly beneficial for edge-device applications such as assistive communication tools or real-time video translation systems. Most recognition errors occur when the signer’s hand is partially out of frame, too close to the camera (causing blur), or when gestures are performed too quickly. These cases suggest that future improvements could include temporal modeling via sequence-based learning or motion smoothing techniques. In addition to quantitative metrics, qualitative inspection of detection outputs confirms that the model can reliably identify hand signs in live video even when multiple signs are performed sequentially. It maintains tight bounding boxes and correct labels across frames, reinforcing its practical applicability. Given its high accuracy, speed, and robustness, YOLOv10-ST is well-suited for real-world use cases such as smart classrooms, mobile apps for the hearing impaired, or sign language translation modules in embedded systems.

### Comparison with transformer-based detectors

To ensure a fair and comprehensive benchmark, we additionally compare the proposed YOLOv10-ST model with state-of-the-art transformer-based object detectors, including DETR^66^, DINO^67^, and ViTDet^68^. These models are evaluated on our ISL dataset or referenced from the literature where applicable. The comparison includes accuracy (mAP), F1-score, and inference speed (FPS), as mentioned in Table 8.

**Table 8:** Comparison with transformer-based detectors.

Model	mAP (%)	F1-score (%)	FPS	Architecture Type
DETR (ResNet-50)	94.8	93.2	7.1	Transformer + CNN
DINO (Swin-L)	96.1	94.8	5.6	Transformer (encoder-decoder)
ViTDet (ViT-L)	96.3	95.1	6.2	Vision Transformer
YOLOv10-ST (Proposed)	**97.62**	**96.58**	**48.7**	Swin

While transformer-based detectors such as DETR, DINO, and ViTDet achieve high accuracy, their inference speeds (5-7 FPS) make them less suitable for real-time ISL recognition applications. YOLOv10-ST achieves higher accuracy (mAP 97.62%) and significantly faster inference (48.7 FPS), making it better suited for deployment in latency-sensitive systems. This demonstrates the effectiveness of our architecture, which combines Swin Transformer’s attention capabilities with YOLOv10’s real-time detection strengths.

### Real-time performance and inference latency

To validate the real-time performance claim of the proposed YOLOv10-ST model, we measured the inference speed and latency under practical conditions. The evaluation was conducted on a system with the following configuration:

- **GPU:** NVIDIA RTX 3080 (10 GB)
- **CPU:** Intel Core i7-12700K @ 3.6 GHz
- **RAM:** 32 GB DDR4
- **Framework:** PyTorch 2.0 with CUDA 11.8
- **Batch size:** 1 (image or video frame)
- **Input resolution:** $$640 \times 640$$

**Performance metrics:**

- **FPS (Frames Per Second):** 48.7 (Images), 45.5 (Video Frames)
- **Average Inference Latency:** 20.5 ms/frame (Images), 22.0 ms/frame (Videos)

These results demonstrate that YOLOv10-ST meets the real-time processing criteria (typically $>30$ FPS or $<33$ms latency per frame). The model is therefore suitable for deployment in time-sensitive ISL applications such as live video translation, assistive communication tools, and mobile sign language apps. **Benchmarking protocol:** All inference speed and latency values were measured using PyTorch 2.0 on an NVIDIA RTX 3080 GPU (10 GB VRAM) and Intel Core i7-12700K CPU, with CUDA 11.8 support. Inference was benchmarked using a batch size of 1 and input resolution of 640$\times$640. For consistency, all YOLO versions (v3 through v10 and YOLOv10-ST) were evaluated under identical conditions using the same model loading, preprocessing, and post-processing pipeline. Latency measurements were averaged over 300 consecutive inferences using Python’s time.time() and confirmed via torch.cuda.synchronize() to ensure synchronization with GPU clock cycles. FPS was computed as the inverse of average latency per frame. These standardized measurements confirm the validity of our real-time performance claims.

### Ablation study: impact of swin transformer and mish activation

To evaluate the individual contribution of Swin Transformer and Mish activation to the overall performance of YOLOv10-ST, we conducted an ablation study. Four variants of the model were trained and tested on the same ISL dataset:

1. Baseline YOLOv10 with default LeakyReLU activation
2. YOLOv10 + Mish activation (no Swin Transformer)
3. YOLOv10 + Swin Transformer (with LeakyReLU)
4. YOLOv10-ST (Full Model: Swin + Mish)

**Table 9:** Ablation study showing the contribution of Swin Transformer and Mish activation.

Model Variant	Swin Transformer	Activation	mAP (%)	F1-score (%)	FPS
Baseline (YOLOv10)	✗	LeakyReLU	$94.87 \pm 0.05$	$93.60 \pm 0.09$	44.6
YOLOv10 + Mish	✗	Mish	$95.23 \pm 0.06$	$94.20 \pm 0.07$	43.8
YOLOv10 + Swin	✓	LeakyReLU	$96.41 \pm 0.04$	$95.10 \pm 0.05$	46.2
YOLOv10-ST (Full)	✓	Mish	${\textbf {97.62}} \pm {\textbf {0.04}}$	${\textbf {96.58}} \pm {\textbf {0.03}}$	**48.7**

To address potential randomness in initialization and training, research repeated each ablation variant for 3 runs with different seeds. The updated Table 9 now includes mean and standard deviation values for both mAP and F1-score. These results confirm that the integration of the Swin Transformer improves average F1-score by approximately 1.5% over the baseline, while Mish activation contributes a more modest 0.6% gain. When combined, the full YOLOv10-ST model improves F1-score by nearly 3.0% over the baseline, demonstrating a synergistic effect. The standard deviations across runs remain under 0.1%, indicating high consistency. This analysis confirms that both components contribute to performance, and their combination yields superior results in ISL recognition tasks.

## Conclusion and future directions

In this paper, a YOLOv10 with Swin Transformer (YOLOv10-ST) model is proposed for both static and dynamic Indian Sign Language Recognition (ISLR). The architecture integrates Swin Transformer blocks into the YOLOv10 framework and incorporates Mish activation to improve feature extraction, attention modeling, and training stability. A custom dataset comprising 15, 000 static images (15 classes) and 35 videos (7 dynamic classes) was used for evaluation. The proposed model demonstrated strong performance with 97.5% precision, 98.1% recall, and 96.58% F1-score on static image data, and 95.24% precision, 96.0% recall, and 95.87% F1-score on video-based dynamic signs.Furthermore, the model achieves real-time inference speeds of 48.7 FPS for images and 45.5 FPS for videos, with an average latency of 20.5 ms per frame, confirming its practical efficiency for real-time ISL applications. The model also achieved a mean Average Precision (mAP) of 97.62% and real-time inference speed of 48.7 FPS, confirming its suitability for real-time applications. Compared to earlier YOLO versions and transformer-based object detectors (e.g., DETR, DINO, ViTDet), YOLOv10-ST outperforms across all key evaluation metrics. The ablation study validates the individual contributions of Swin Transformer and Mish activation, while paired t-tests confirm statistical significance (p $< 0.005$). While the proposed model demonstrates strong performance across static and dynamic signs, we acknowledge that the video dataset currently contains a limited number of samples (35videos across 7 classes). Although frame-wise decomposition and spatial modeling partially address this, expanding the video portion of the dataset remains a priority. Future work will focus on collecting a larger, more diverse set of dynamic ISL gestures, including multi-signer, multi-background, and continuous signing data, to further validate the model’s generalizability in real-world applications. This study currently focuses on architectural and performance improvements; however, we acknowledge the need for explainability in real-world use cases. In future work, we aim to incorporate Grad-CAM and Swin Transformer attention visualizations to interpret model focus areas during sign classification. This will be especially important for clinical, educational, and interactive deployment scenarios where model transparency is essential. We acknowledge the importance of formal ethics protocols. While the current dataset was collected with informed verbal consent and anonymized handling, future versions of this work will incorporate written consent forms and formal institutional ethical approval to align with international research ethics standards.

## Data Availability

The dataset used in this study, consisting of 15,000 static ISL images and 35 videos across 7 dynamic sign classes, was collected privately with participant consent. Due to ethical restrictions and lack of public consent release at the time of collection, the dataset is not publicly hosted. However, researchers may request access to the dataset by contacting the corresponding author and signing a data usage agreement. This process ensures that the dataset is used solely for academic and non-commercial research purposes while complying with participant privacy and institutional guidelines.
